# Extracellular Matrix Molecular Remodeling in Human Liver Fibrosis Evolution

**DOI:** 10.1371/journal.pone.0151736

**Published:** 2016-03-21

**Authors:** Andrea Baiocchini, Claudia Montaldo, Alice Conigliaro, Alessio Grimaldi, Virginia Correani, Francesco Mura, Fabiola Ciccosanti, Nicolina Rotiroti, Alessia Brenna, Marzia Montalbano, Gianpiero D’Offizi, Maria Rosaria Capobianchi, Riccardo Alessandro, Mauro Piacentini, Maria Eugenia Schininà, Bruno Maras, Franca Del Nonno, Marco Tripodi, Carmine Mancone

**Affiliations:** 1 National Institute for Infectious Diseases L. Spallanzani, IRCCS, via Portuense 292, 00149 Rome, Italy; 2 Department of Cellular Biotechnologies and Haematology, Sapienza University of Rome and Istituto Pasteur - Fondazione Cenci Bolognetti, Via Regina Elena 324, 00161 Rome, Italy; 3 Dipartimento di Scienze Biochimiche, Sapienza University of Rome, P.le Aldo Moro 5, 00185 Rome, Italy; 4 Dipartimento di Chimica, Sapienza University of Rome, P.le Aldo Moro 5, 00185 Rome, Italy; 5 Dipartimento di Biopatologia e Biotecnologie Mediche, University of Palermo, Via Divisi 83-90133, Palermo, Italy; 6 Institute of Biomedicine and Molecular Immunology (IBIM), National Research Council of Italy, Palermo, Italy; 7 Department of Biology, University of Rome 'Tor Vergata', Rome, Italy; University of Navarra School of Medicine and Center for Applied Medical Research (CIMA), SPAIN

## Abstract

Chronic liver damage leads to pathological accumulation of ECM proteins (liver fibrosis). Comprehensive characterization of the human ECM molecular composition is essential for gaining insights into the mechanisms of liver disease. To date, studies of ECM remodeling in human liver diseases have been hampered by the unavailability of purified ECM. Here, we developed a decellularization method to purify ECM scaffolds from human liver tissues. Histological and electron microscopy analyses demonstrated that the ECM scaffolds, devoid of plasma and cellular components, preserved the three-dimensional ECM structure and zonal distribution of ECM components. This method has been then applied on 57 liver biopsies of HCV-infected patients at different stages of liver fibrosis according to METAVIR classification. Label-free nLC-MS/MS proteomics and computation biology were performed to analyze the ECM molecular composition in liver fibrosis progression, thus unveiling protein expression signatures specific for the HCV-related liver fibrotic stages. In particular, the ECM molecular composition of liver fibrosis was found to involve dynamic changes in matrix stiffness, flexibility and density related to the dysregulation of predominant collagen, elastic fibers and minor components with both structural and signaling properties. This study contributes to the understanding of the molecular bases underlying ECM remodeling in liver fibrosis and suggests new molecular targets for fibrolytic strategies.

## Introduction

The extracellular matrix (ECM) is a critical component of the human liver microenvironment. Forming a fibrous scaffold, the ECM provides a surface for cell adhesion, space for cell growth and migration, and acts as reservoir for signaling molecules [1]. Moreover, several ECM components, such as collagen, fibronectin and laminin, are responsible also for promoting the expression of liver-specific functions and cell differentiation [2]. Furthermore, the local stiffness of the liver ECM is an important mechanical effector of cellular behavior and tissue formation. Particularly, cellular responses to mechanical signals of liver ECM include differentiation, migration, proliferation, and alterations in cell—cell and cell—matrix adhesion [3].

Wound repair is a dynamic process in which the liver ECM composition and stiffness become critically important. In fact, continuous ECM remodeling during chronic liver injuries leads to an altered and excessive accumulation of extracellular proteins, proteoglycans and carbohydrates, thus leading to fibrosis, which is responsible for the morbidity and mortality associated with liver failure [4]. Therefore, the identification and quantitation of the ECM components, spatial and temporal dynamics of ECM molecules, and interactions underpinning ECM proteins networks represent the key steps towards understanding the role of ECM in tissue remodeling in liver disease. However, the study of human liver ECM components is generally hampered by the difficulty of isolating the matrix scaffold from hepatic cells.

Liver decellularization by portal perfusion is an attractive technique for the whole scaffold isolation. In animal models, this approach allows for isolating the ECM, retaining the architecture of the original tissue and permitting applications, both in large-scale investigations of components and in tissue engineering for regenerative medicine [5]. However, human liver decellularization by portal perfusion is only applicable in explanted diseased organs, thus limiting any study at the onset and progression of liver fibrosis.

To date, the proteomic attempts to characterize the components of human liver ECM were performed by analyzing the secretion of ECM producing cells *in vitro* or by protein enrichment from homogenized liver tissues [6, 7]. However, cell-derived ECM is a far cry from reflecting the native liver scaffold and does not allow for any study on the ECM dynamics in liver fibrosis progression. On the contrary, studies based on the ECM protein enrichment from homogenized liver tissues do not allow for reducing or eliminating the contribution of cellular components, hence, resulting in overestimations of quantitative data acquired by proteomic approaches. Thus, deciphering the human ECM molecular composition from isolated liver scaffolds still represents an important scientific challenge.

Here, we designed a biochemical-based strategy for the isolation of human hepatic ECM scaffolds that is already effective on liver biopsy specimens. This decellularization approach allows for preserving the “bonafide” native matrix structure and, net of cell membrane ECM-interacting proteins (e.g. integrins, ADAMTS, MT-MMPs), the ECM molecular composition. Then, we made use of the isolated liver scaffolds to delineate and compare, by a label-free quantitative mass-spectrometry-based approach, the proteome changes of ECM components in the different stages of liver fibrosis in HCV-infected patients. This investigation first discloses the ECM molecular composition that characterizes the transition from moderate to severe fibrosis, and defines a specific ECM signature for the bridging fibrosis with nodular regeneration (cirrhosis).

## Materials and Methods

### Ethics statements and samples

The collection and use of residual diagnostic liver biopsy samples for research purposes has been approved by the ethical committee of the “L. Spallanzani” National institute for Infectious Diseases, IRCCS, Rome, Italy. The patients accepted to participate in the study by signing an informed consent. All the samples have been completely anonymized, leaving only clinical information relevant for study purposes.

The collection and use of murine liver samples has been approved University of Palermo Animal Care Committee. Murine liver specimens were harvested from 6-week-old mice (Charles River Laboratories), maintained in accordance with the institutional guidelines of the University of Palermo Animal Care Committee. All mice were housed under controlled conditions for temperature and humidity in a specific pathogen free facility, using a 12 h light/dark cycle and ad libitum access to water and food.

The experimental procedures have been communicated to the Ministry of Health in accordance with the ministerial directive at the time of the experiments: legislative decree 116/92, annex 4 to the circular of the Ministry of Health n. 8 of 1994. No suffering was inflicted on the animals before euthanasia. Mice welfare was evaluated by checklist for the determination of endpoints: appearance, natural behavior, hydration status, clinical signs, provoked behavior, grimace scale and body conditioning scoring. No animals became severely ill or died prior to the experimental endpoint. Euthanasia in mice was performed by carbon dioxide asphyxiation.

### Liver tissue decellularization

Plasma proteins removal from liver tissues was performed by overnight shaking (600 RPM) in Step 1 Washing Buffer (0,5 M NaCl, 10 mM Tris Base pH 7.5, 1X protease inhibitor) at 4°C. After centrifugation at 13000 RPM for 1 minute, the supernatants (plasma proteins) were stored at −80°C. Pellets were washed twice with Step 2 Decellularization Buffer (DB) (1% SDS in PBS, 1X protease inhibitor) and incubated overnight in DB at room temperature, shaking at 800 RPM. On the next day, the supernatants were removed and stored at −80°C and fresh DB was added to the tissues. This process was repeated until the tissues were completely decellularized (two to three days for bigger specimens). All supernatants from the Step 2 process (containing mainly cellular proteins) were pooled in one tube. Decellularized tissues were washed twice with deionized water and incubated with 80% acetone for 90 minutes to remove residual SDS. After centrifugation at 12000 RPM for 15 min at 4°C the supernatants were discarded and the ECM scaffold samples were washed twice with PBS.

### Histological analyses

Each human biopsy sample was evaluated histologically according to the METAVIR grading system for disease stage and grade of necroinflammatory activity. Tissue specimens were fixed in 10% buffered formalin and embedded in paraffin. 4μm-thick slices were cut and stained with hematoxylin and eosin (H&E), Masson’s trichrome and silver impregnation. Immunostaining was performed on Benchmark XT system (Ventana, Tucson, AZ, USA). Fibronectin (BD Biosciences, Erembodegem, Belgium), albumin and E-cadherin (Abcam, Cambridge, UK) antibodies were used at dilution of 1:200. Liver lobes from mouse explants, after decellularization, were stained with DAPI over night and observed at inverted microscope Nikon Eclipse Ti.

### Scanning Electron Microscopy, Western blotting, Mass spectrometry and data analysis

See S1 File for more detail.

## Results

### Liver ECM scaffold isolation by tissue decellularization

ECM isolation by immersion and agitation in decellularization agents has been applied for a wide variety of tissue specimens, including dermis, skeletal muscles, peripheral nerves and heart valves [8]. However, the particular hepatic tissue cellularity, density and thickness have been critical determinants that hampered the translation of this approach to liver biopsy specimens [8].

**Fig 1:**
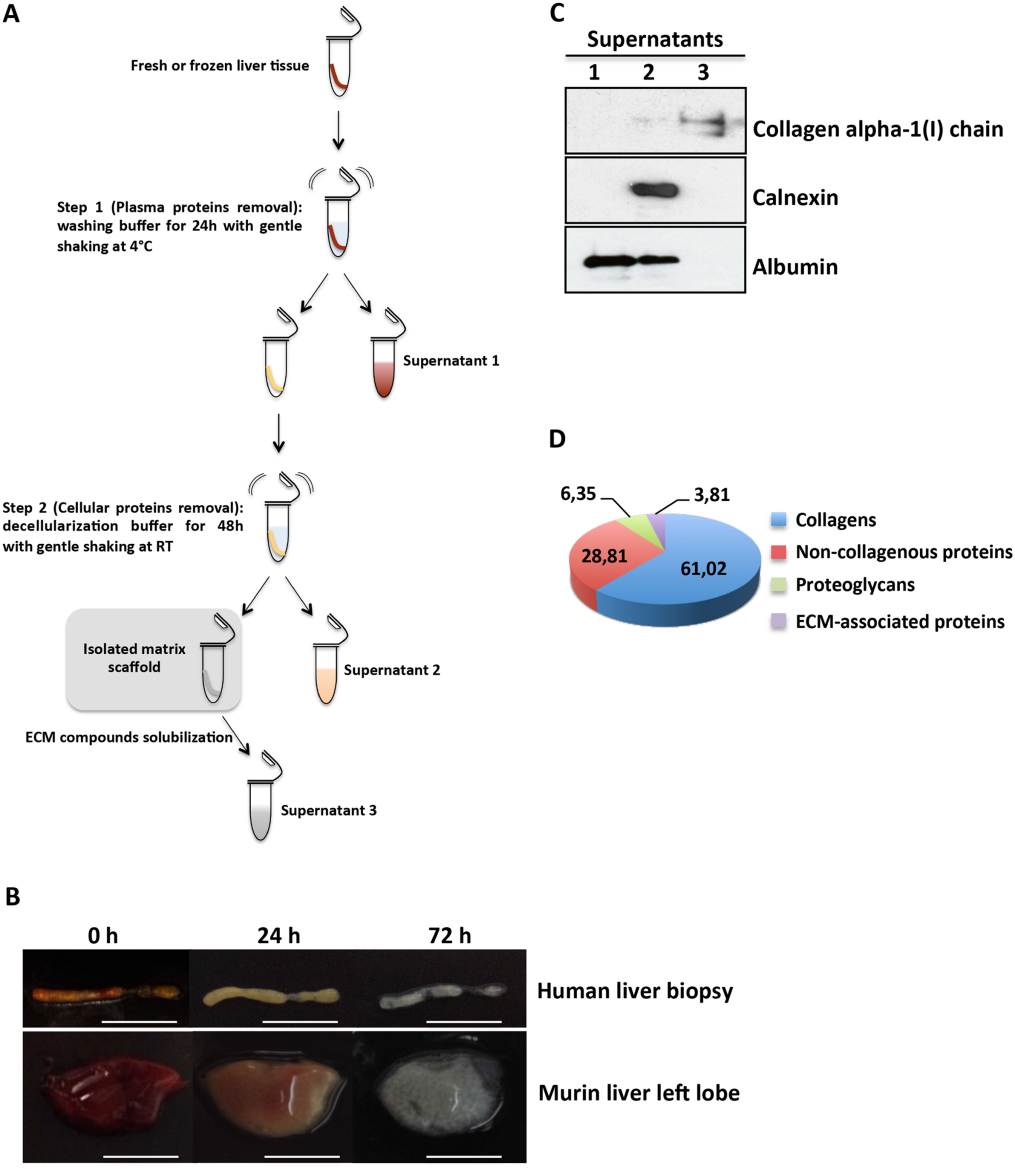
Decellularization of liver tissues. (A) Schematic representation of the two-step decellularization protocol; for details see Methods section. (B) Representative images of human and murine liver tissues during the decellularization process at the indicated times. Scale bars: 5 mm. (C) Western blot analysis of supernatants for the indicated proteins. For each gel lane, 10 μg of protein sample were loaded; albumin was used as plasma and cellular protein markers; calnexin was used as a cellular protein marker. The lack of calnexin in supernatant 3 indicates the removal of plasma and cellular proteins from the scaffold. One representative experiment out of three is shown. (D) Percentage of ECM category components of human liver scaffold. The pie chart displays the results of one human decellularized liver biopsy with low fibrosis processed through the proteomics workflow. For each identified protein, protein abundance was calculated using the frequency of tandem mass spectra assigned to that protein.

We achieved human liver ECM scaffold isolation by establishing a two-step protocol that requires a liver biopsy immersed in differential buffers and then subjected to gentle and continuous agitation (Fig 1A). In the first step, the blood was flushed out from the liver biopsy by means of a washing buffer, thus turning the color of the tissue to yellowish-brown in 24 hours (Fig 1B, upper panels). In the second step, after 48 hours of decellularization buffer, a translucent acellular scaffold, which retained the gross shape of a liver biopsy, was generated (Fig 1B, upper panels). Notably, similar results were obtained applying this protocol to mouse liver lobes, thus indicating that the effectiveness of the method is not limited to the biopsy specimens size (Fig 1B, lower panels). Western blotting analysis provided evidence of the successful isolation of liver bioscaffold highly enriched for ECM components (Fig 1C). Mass spectrometry analysis highlighted that collagens represented more than 60% of the human liver ECM scaffold molecules, while non-collagenous proteins and proteoglycans contributed to 28,81% and 6,35%, respectively. (Fig 1D and S1 Table). As expected, the analysis of total collagen components showed a predominant abundance of fibrillar collagen type I (COL1A1 and COL1A2) (S1 Fig). Interestingly, five of the collagen proteins (COL2A1, COL21A1, COL23A1, COL5A3 and COL26A1) were found in the matrix of human adult liver. To the best of our knowledge, this study represents the first evidence of these proteins in the matrix of human adult liver, thus further highlighting the efficacy of the ECM scaffold isolation procedure.

### Characterization of the decellularized liver ECM scaffold

**Fig 2:**
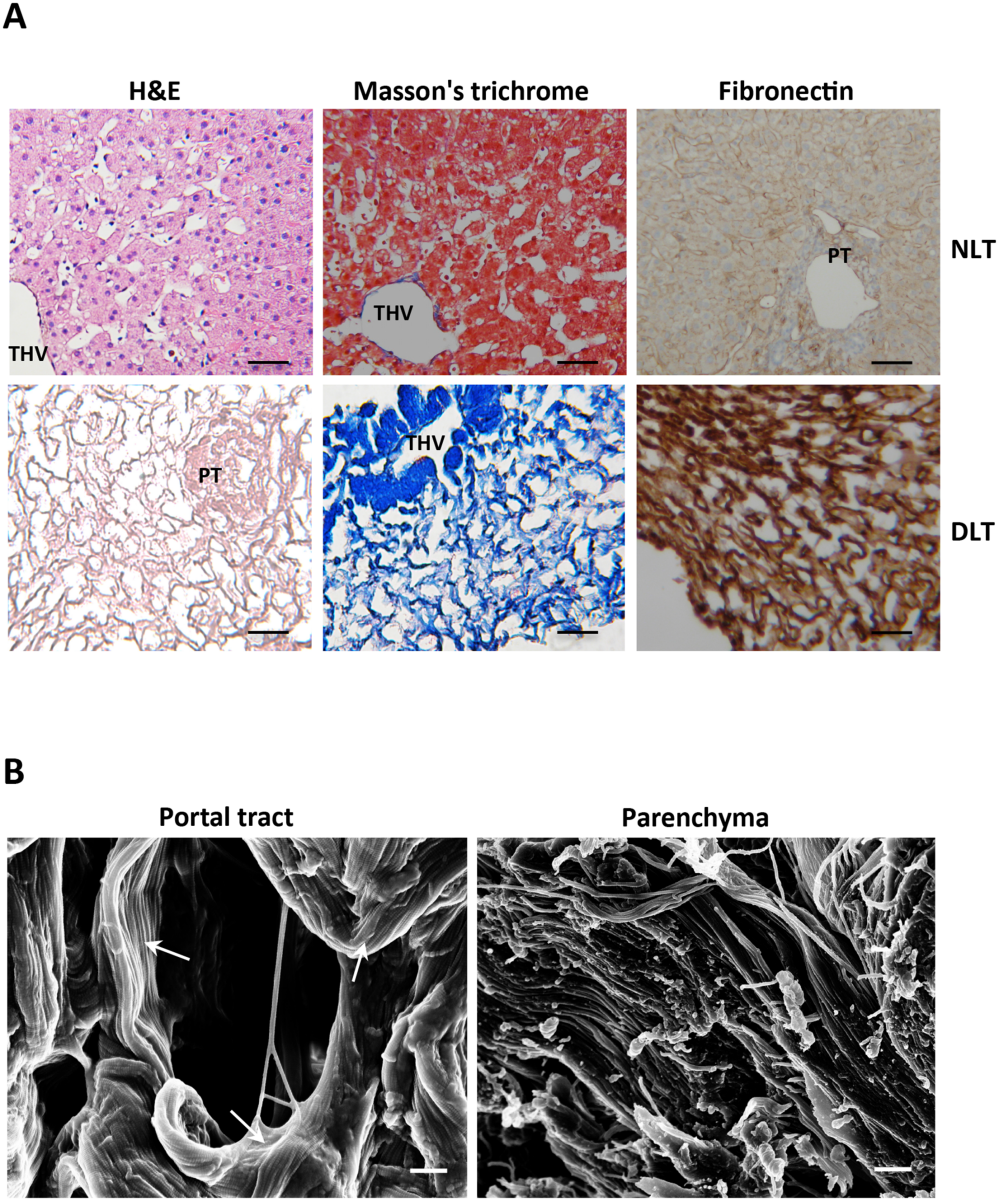
Histological and ultrastructural analysis of decellularized human liver ECM scaffold. (A) Comparison of human normal liver (NLT) and decellularized liver (DLT) tissues for the indicated staining. Portal tracts (PT) and terminal hepatic veins (THV) are indicated. Scale bars: 100 μm. (B) SEM images of the extracellular matrix in the portal tract and parenchyma. Collagen fibers (white arrows) are indicated. Scale bars: 1 μm.

To investigate the effects of decellularization protocol on the extracellular matrix network, histological investigations were performed. Hematoxylin/eosin and trichrome stain highlighted that no nuclei or cytoplasmic staining were observed in decellularized human liver tissue (Fig 2A, left and middle panel respectively), as well as the immunostaining for fibronectin showed that the isolated liver ECM scaffold retained the typical network of web-like structures (Fig 2A, right panel). Moreover, the portal tracts and terminal veins connective structures were preserved, suggesting that the microvascular scaffold remained intact (Fig 2A). Notably, the same results were observed when the decellularization process was applied to larger portions of liver tissue (i.e. murine hepatic lobe); S2 Fig shows as the scaffold of the vascular tree is clearly apparent, thus suggesting that the larger circulation system of vessels was also preserved.

It is well established that normal liver parenchyma contained only few collagen fibers, while these are more abundant in the portal tract [9]. By means of scanning electron microscopy (SEM), we further investigated if our decellularization approach was able to retain the different zonation of collagen structures. As shown in Fig 2B, we found that the density of collagen fibers was higher in the portal tracts than in the parenchymal area, which instead appears as a non-fibrillar network structure.

Taken together, these data demonstrated that this decellularization method generates hepatic scaffolds that preserve the main structural properties of human ECM, thus filling a void in the availability of proper experimental tools for studying human liver biology.

### Molecular remodeling of human ECM in HCV-induced liver fibrosis

Fifty-seven decellularized liver biopsies were processed to evaluate the molecular ECM changes occurring during fibrosis progression in HCV-infected patients. By histological diagnosis, samples of 1 cm in length were classified according to the Metavir score as follows: 14 in portal fibrosis (F1); 19 in moderate fibrosis (F2); 14 in severe fibrosis (F3); and 10 in bridging fibrosis (F4) (S2 Table). Liver samples were then individually decellularized, and the produced ECM liver scaffolds underwent trypsin digestion. The obtained peptides were pooled according to fibrotic stage and then subjected to proteomic analysis.

**Fig 3:**
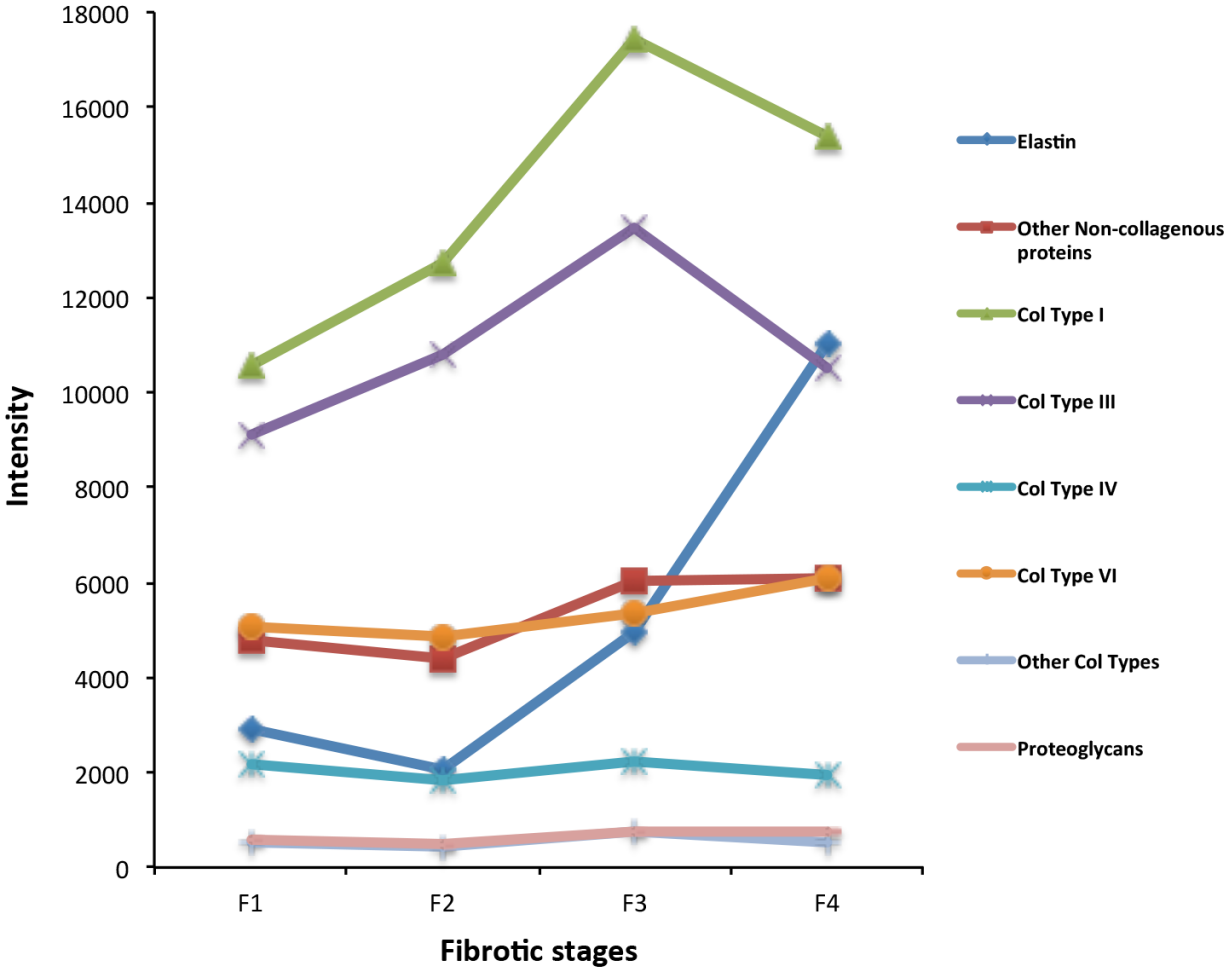
Dynamic changes of the major ECM structural components in HCV-induced liver fibrosis progression. Protein abundance was calculated using the LTQ intensity assigned to that protein. LTQ intensity was determined as average of three independent experiments. For details see S2 Table.

By means of the nLC-MS-based label free proteomics approach, we quantified forty-seven ECM-associated proteins to be differentially expressed in the evolution of liver fibrosis. Firstly, we analyzed the absolute abundance of the major structural ECM components. As expected, liver fibrosis evolution from F1 to F3 stages requires a progressive accumulation of fibrillar collagen (type I and III) and elastin (Fig 3 and S2 Table). Interestingly, the achievement of liver cirrhosis (F4) involves a massive accumulation of elastic fiber (i.e. elastin overexpression in Fig 3) concomitantly with a moderate decrease of type I and III collagens. These data suggest that the liver fibrosis progression modifies the liver stiffness by a combination of predominant collagen and elastic fibers.

**Fig 4:**
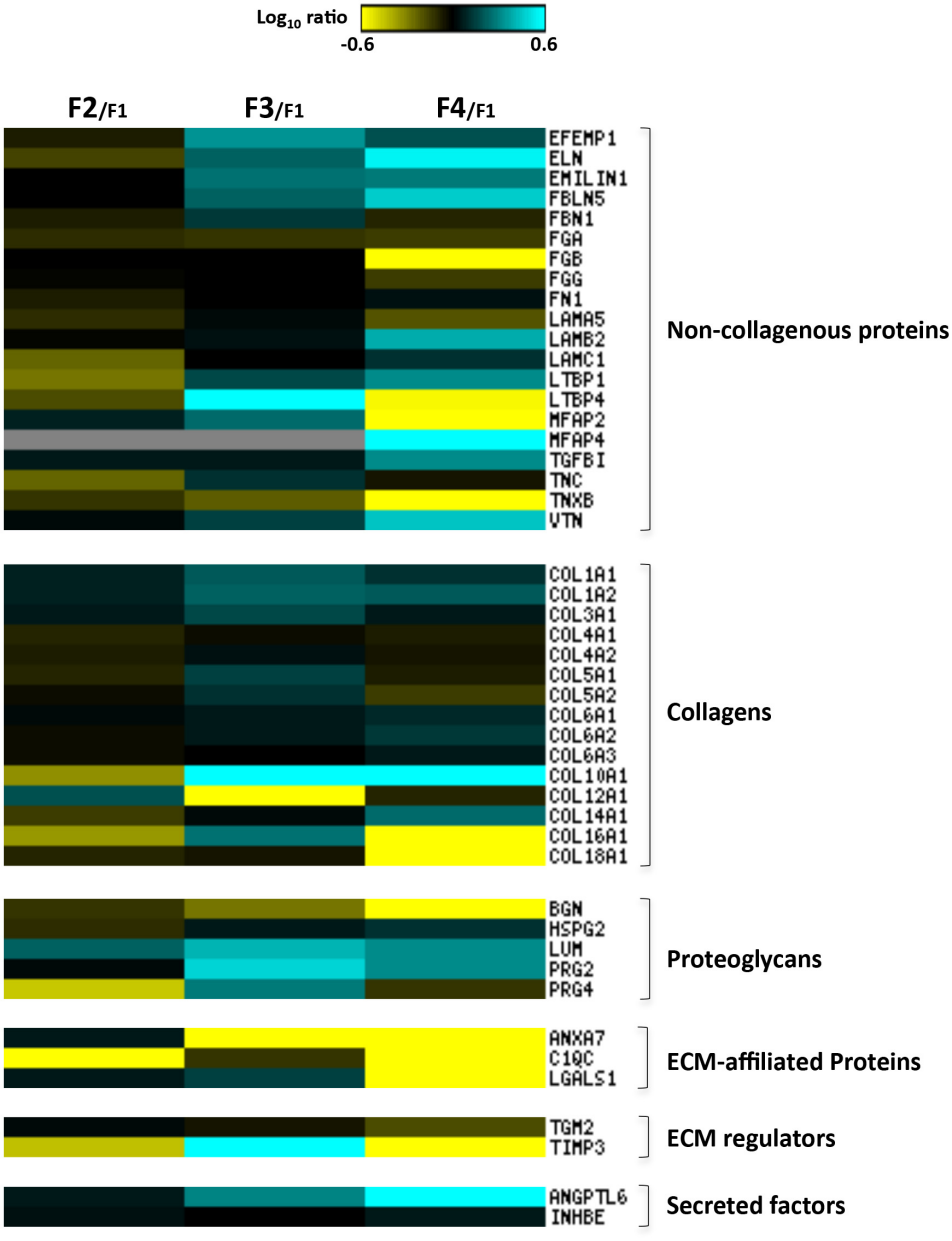
Liver ECM proteome profiles in HCV-induced liver fibrosis progression. Abundance ratios F2/F1, F3/F1 and F4/F1 were calculated and analyzed by heat map analysis. Each vertical column represents an individual abundance ratio and each horizontal row an individual protein. Protein abundance ratios were colored according to fold changes (yellow Log10 ratios: down-regulations; blue Log10 ratios: up-regulations), and the color scale indicates the magnitude of expression changes. Black squares indicate no change in protein abundance. Gray squares indicate missing data.

Then, to clarify the specific contribution of each ECM component in liver fibrosis progression, a relative abundance analysis was performed by comparing the protein expression profiles of F2, F3 and F4 stages in respect to the minimal stage (F1) (Fig 4 and S2 Table). By heat map analysis (Matrix2png, version 1.2.2), we unveiled that fibrotic stage F2 involves a slight increase for the most predominant collagens (type I and III) (Fig 4). Among the lower abundance collagen chains, COL10A1, COL14A1, COL16A1 were down-regulated, while COL12A1 was found up-regulated. Surprisingly, a general down-regulation of non-collagenous proteins, such as elastin (ELN), tenascin (TNC), laminin gamma-1 (LAMC1), latent-transforming growth factor beta-binding proteins 1 (LTBP1) and 4 (LTBP4) was observed. Among the proteoglycans, proteoglycan 4 (PRG4) was down-regulated; on the contrary, lumican (LUM) expression was found increased. Interestingly, metalloproteinase inhibitor 3 (TIMP3), known to irreversibly inactivate the collagenases, and to be a critical regulator of hepatic inflammation, cell death and survival [10, 11], was found underexpressed (ratio F2/F1 = 0,36), thus suggesting a tissue response to the pathological accumulation of ECM.

Severe fibrosis (F3) requires the overexpression of most of the ECM protein components (Fig 4). As expected, type I and III collagen levels and the most abundant non-collagenous proteins (ELN, fibulin-3 (EFEMP1), fibulin-5 (FBLN5), emilin-1) were found up-regulated. These data are consistent with the strong increase in TIMP3 expression (ratio F3/F1 = 4,59), thus indicating the inhibition of fibrolytic processes. Interestingly, protein abundances of COL10A1, COL12A1, COL16A1, LTBP1 e LTBP4 showed opposite trends in respect to those observed in the F2 stage. With the exception of biglycan (BGN) and perlecan (HSPG2), the expression levels of proteoglycan components (LUM, PRG2, PRG4) were found increased two- to three-fold.

Achievement of bridging fibrosis (F4) with nodule formation (cirrhosis) is characterized by a protein expression pattern that differs significantly from the F3 stage. As expected, the expression levels of ELN, FBLN5, microfibril-associated glycoprotein 4 (MFAP4) and vitronectin (VTN) were detected with a minimum three-fold increase. On the contrary, several non-collagenous proteins such as fibrinogen beta chain (FGB), LTBP4, microfibrillar-associated protein 2 (MFAP4), tenascin-X (TNXB) were found underexpressed, with expression levels 5 times lower. Predominant collagen profile is characterized by a slight transition from the type I, III and V fibril collagen to the non-fibril, network-forming type VI. Notably, in respect to stage F3, the lower abundance collagen chains expression showed COL14A1 in overexpression and COL16A1 and COL18A1 strongly decreased. Proteoglycan expression revealed the up-regulation of LUM and PRG2, while the abundance levels of BGN, that showed a gradual decrease during fibrosis progression, were found underexpressed up to 12 times in cirrhosis. Interestingly, the transition from stage F3 to F4 involves the down-regulation of all the identified ECM-affiliated proteins and ECM regulators. Finally, angiopoietin-related protein 6 (ANGPTL6), a secreted protein known to be involved in the wound healing process by promoting remodeling and regeneration of tissues [12], was found strongly up-regulated.

**Fig 5:**
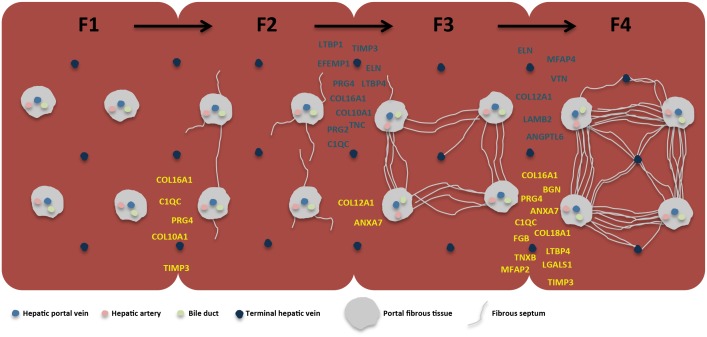
Schema summarizing the ECM protein components that are differentially regulated in the transitions among the fibrotic stages. Data are shown as follows: blue for up-regulated proteins, yellow for down-regulated proteins; the position in the graph indicates the transition between two fibrotic stages in which the protein was found differentially regulated.

Finally, in order to focus on ECM proteins that may represent a molecular signature of transition from one stage to another the protein ratios of F2/F1, F3/F2 and F4/F3 were calculated by the ratio of the ratios and the resulting values converted in fold changes (S3 Table). Then, proteins with a higher fold-change (>2) and lower fold-change (<2) abundances were considered and are represented in Fig 5. Interestingly, we found that most of the dysregulated proteins were the so far undescribed minor components of the ECM (listed in S3 Table and Fig 4), thus indicating that the transitions among the HCV-related fibrous stages involve fiber formation and accumulation, as well as dynamic changes in molecules with signaling properties.

## Discussion

Obtaining a comprehensive description of the ECM molecular composition in human liver health and disease has so far been hampered by the difficulty to isolate decellularized hepatic ECM scaffolds. This study first describes the isolation of liver tissue-derived ECM scaffolds that preserve the “bona fide” three-dimensional architecture and native matrix composition.

Then, once the suitability of the decellularization protocol was verified, we made use of it to attempt to make a comprehensive study of human ECM dynamic changes in the course of HCV-induced liver fibrosis. New fibrotic stage-specific molecular signatures have been unveiled, in particular, highlighting the involvement of the previously described minor components of the ECM.

To date, liver ECM scaffold isolation is based on portal vein perfusion of whole animal organs [5, 13], while decellularization of specimens (i.e. biopsy and surgical samples) have so far been, hampered by hepatic tissue cellularity, density and thickness. To circumvent this issue, biochemical and mechanical conditions to sequentially remove the circulating and cellular fractions from the liver tissue were developed. The resulting hepatic matrix shows translucent scaffolds, zonal distribution of ECM components, and suggests intact micro- and macrovascular trees similar to those obtained by perfusion-based whole organ decellularization. In light of this, the proposed protocol, that is independent from the perfusion, may justify efforts to scale up the technique for applications in humans.

The development of liver fibrosis, particularly cirrhosis, is associated with significant morbidity and mortality. At present, since the only curative treatment for end-stage cirrhosis is transplantation, there is a considerable need for therapeutic strategies to counteract fibrosis progression. A prerequisite for developing such strategies is having ample knowledge of ECM-associated molecules. This study, performed directly on purified hepatic ECM scaffolds, allowed for a comprehensive and specific analysis of the liver ECM components. The identification of COL2A1, COL21A1, COL23A1, COL5A3 and COL26A1 as new molecular components of the liver ECM confirms this.

With respect to the final and most ambitious goal guiding our research, to compare the ECM proteome changes occurring in all the fibrotic stages of HCV-infected patients, overall, the obtained data i) are in good agreement with the changes observed by recent reports, ii) unveil new fibrotic stage-specific molecular signatures, iii) underline the molecular bases of the METAVIR score system, thus confirming its efficacy.

In fact, while fibrotic stage-specific changes of several ECM components (among them LUM, FBLN5, COL14A1, MFAP4, VTN, COL6A1) are in line with recent reports [14–16], new data highlight the dysregulation of non-fibrillar collagens, proteoglycans and additional minor components of the liver ECM.

### (F2)

With the exception of a slight increase for collagen types I and III, the transition from portal to moderate fibrosis is characterized more by ECM remodeling than by its increase. In particular, a general down-regulation of the FACIT (fibril-associated with interrupted triple helics) collagens (COL14A1 and COL16A1) was observed. Since FACIT collagens, associating with mature portal fibrils of collagen I and III, confer flexibility to them [17], our data suggest that the ECM in moderate fibrosis acquires a dense structure. The low flexibility of the portal ECM in moderate fibrosis is further suggested by the down-regulation of PRG4, also called lubricin, known for its lubricating and protective actions in many connective tissues, including the liver [18].

### (F3)

The main efforts to manage HCV-related liver disease are concentrated in developing anti-fibrotic strategies to counteract the transition from moderate to severe fibrosis. In fact, as fibrosis progresses to the F3 stage, portal to central gradients are lost, septa become continuous, and resident cells in the space of Disse dedifferentiate [4]. Here, we observed that all of the most abundant ECM components (i.e. fibrillar collagens, proteoglycans and fibronectin) were up-regulated, thus confirming that the hepatic ECM acquires stiffness.

Massive ECM accumulation is normally induced by the ECM-resident profibrogenic molecules. The transforming growth factor-b (TGF-b) is one of the major profibrogenic molecules: its overexpression has been demonstrated to induce severe liver fibrosis [19]. TGF-b is secreted to the extracellular space in the liver as a latent complex bound to latent TGF-b-binding proteins (LTBPs) [20]. LTBP1 was previously found to be up-regulated in liver tissue from patients with chronic hepatitis C [21].

Notably, large amounts of two members of LTBPs (LTBP1 and LTBP4) were found deposited in the ECM of patients with stage F3 fibrosis. In light of this, it is conceivable that LTBP1- and 4-mediated deposition, and targeting of latent, activatable TGF-beta into ECM may exert a pivotal role in F2-F3 transition. We believe that the involvement of these two cytokines in severe fibrosis needs to be addressed in further studies.

### (F4)

The entire liver architecture is lost when fibrosis advances to cirrhosis. This is generated by the formation of massive dense fibrous septa that delineate hepatocellular nodules [22]. This study unveiled that the ECM composition of the cirrhotic liver shows a characteristic molecular signature for the non-fibrillar collagenous components. In particular, our data support the hypothesis that cirrhotic-specific ECM remodeling may be mediated by the overexpression of proteins involved in network-forming collagen and elastic fiber assembly. COL6A1 is the main component of the heterotrimeric collagen VI, which forms branching filamentous networks [23]. Once in overexpression, it anchors structures, such as the blood vessels, to fibrillar collagens, thus distorting the tissue architecture [24]. The elastic fibers are formed from elastin and many elastic microfibril proteins (consisting of numerous proteins such as microfibrillar-associated glycoproteins, fibrillins, fibullins and elastin receptors) [25]. Increased amounts of elastic fibers have been associated to the pathological remodeling of tissue [26]. Moreover, fibrous septa particularly rich in cross-linked elastic fibers are known to be more resistant to matrix metalloproteinases (MMPs)-mediated degradation, thus contributing to fibrosis irreversibility [27]. Here, a massive accumulation of ELN, FBLN5 and MFAP4 in the ECM of cirrhotic patients was observed. Therefore, these proteins might represent multiple targets to develop therapeutic strategies to facilitate liver cirrhosis reversion. Fig 5 recapitulates the HCV-associated liver fibrotic molecular stage-specific hallmarks observed here, and notably, emphasizes the match between the METAVIR-based histological evaluation and the molecular dynamic changes observed.

In conclusion, this study contributes to the knowledge of human ECM composition in liver fibrosis progression. However, we believe that this should be considered as a starting point, as many issues on the ECM structure of fibrotic liver still remain to be addressed. What is the intra- and intermolecular cross-linking profile? What cross-links have a role in inhibiting ECM degradation? What is the cellular origin of the described proteins? What kind of post-transcriptional and post-translational regulations occur in the pathological deposition of the ECM molecules? We believe that the presented data will support future studies to address these issues.

## Supporting Information

S1 Fig Supplementary Fig 1. Percentage of collagen chains (A) and non collanegenous proteins (B) in a human liver ECM scaffold.
(PDF)

S2 Fig Supplementary Fig 2. Histological analysis of decellularized murine hepatic lobe.
(PDF)

S1 File Supplementary Materials and Methods. (DOCX)

S1 Table Supplementary Table I. Proteomic Results from one human decellularized liver biopsy with low fibrosis.
(XLS)

S2 Table Supplementary Table II. Clinicopathological features of Fibrotic Patients and Label free proteomic analysis.
(XLSX)

S3 Table Supplementary Table III. Protein abundance ratios of F2/F1, F3/F2 and F4/F3 fibrotic stages.
(XLSX)

## Abbreviations

ECM: extracellular matrix
nLC-MS: nano liquid chromatography-mass spectrometry
SEM: Scanning Electron Microscopy
NLT: normal liver tissue
DLT: decellularized liver tissue
PT: portal tracts
THV: terminal hepatic veins
ADAMTS: a disintegrin and metalloproteinase with thrombospondin motifs
MT-MMPs: membrane-bound MMPs (MT-MMPs)
