# Supplementary material for: Extracellular Matrix Molecular Remodeling in Human Liver Fibrosis Evolution
Source: PLoS One. 2016 Mar 21;11(3):e0151736. doi: 10.1371/journal.pone.0151736 (PMC4801190; doi:10.1371/journal.pone.0151736)
Supplement: S1 Fig — Percentage of collagen chains (A) and non collanegenous proteins (B) in a human liver ECM scaffold. (PDF) [file pone.0151736.s001.pdf]

Supplementary Figure 1

A

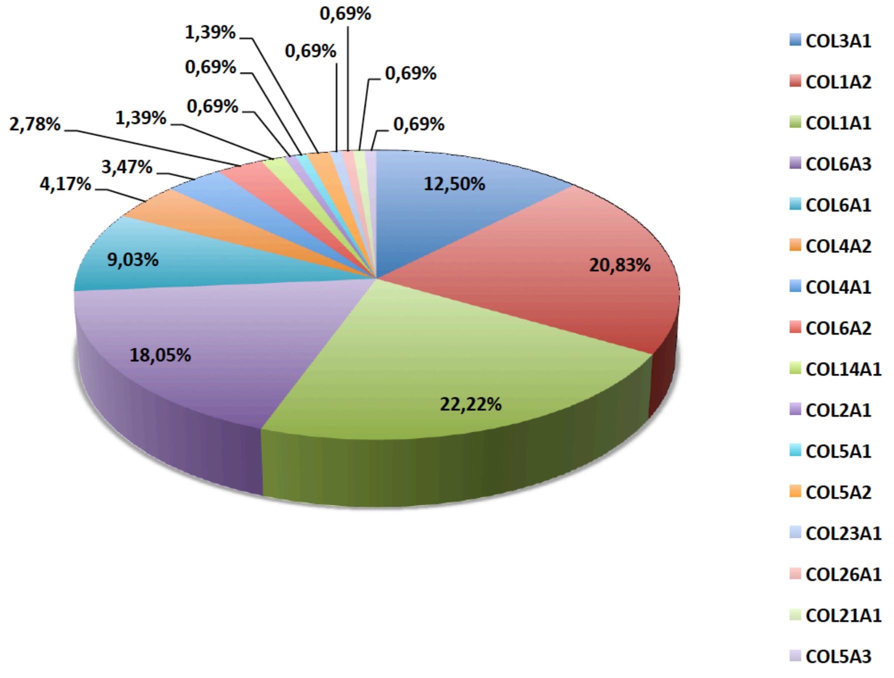

B

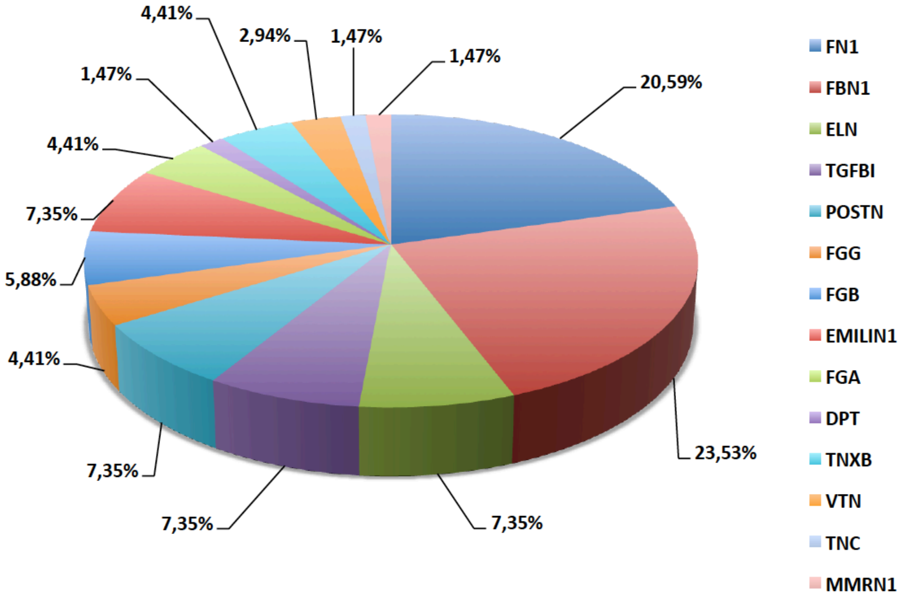

Percentage of collagen chains (A) and non-collanegenous proteins (B) in a human liver ECM scaffold. The pie charts display the results from one human decellularized liver biopsy with low fibrosis processed through the proteomics workflow. For each identified protein, protein abundance was calculated using the frequency of tandem mass spectra assigned to that protein.
