# Supplementary material for: Extracellular Matrix Molecular Remodeling in Human Liver Fibrosis Evolution
Source: PLoS One. 2016 Mar 21;11(3):e0151736. doi: 10.1371/journal.pone.0151736 (PMC4801190; doi:10.1371/journal.pone.0151736)
Supplement: S2 Fig — Histological analysis of decellularized murine hepatic lobe. (PDF) [file pone.0151736.s002.pdf]

Supplementary Figure 2

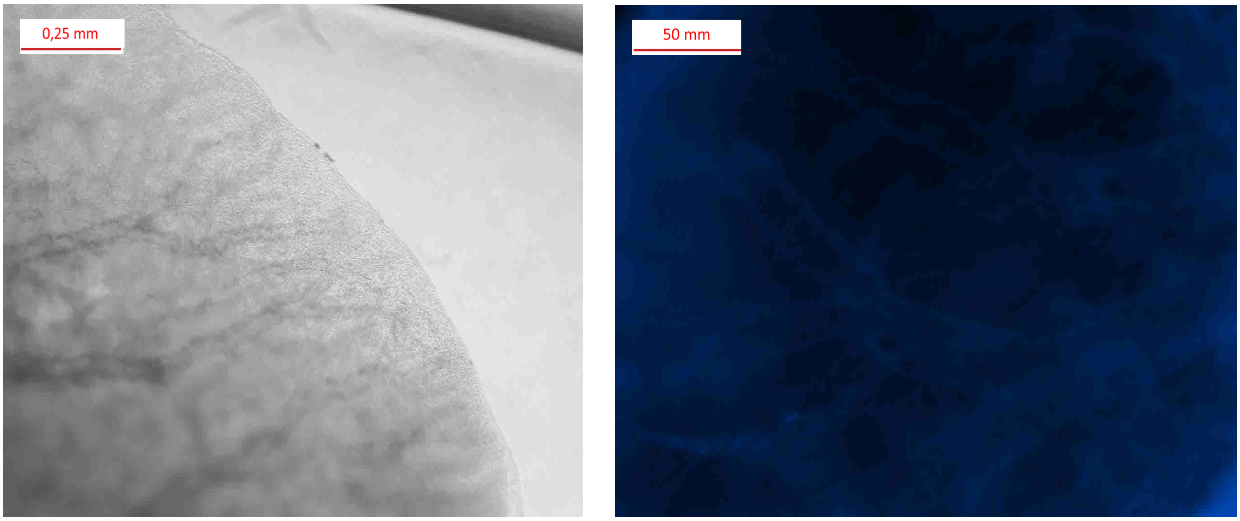

Histological analysis of decellularized murine hepatic lobe. Left panel: phase contrast of decellularized liver ECM scaffold. Right panel: decellularized liver stained with DAPI, no nuclei were detected. Scal bar indicated in the pictures.
