# Supplementary material for: Extracellular Matrix Molecular Remodeling in Human Liver Fibrosis Evolution
Source: PLoS One. 2016 Mar 21;11(3):e0151736. doi: 10.1371/journal.pone.0151736 (PMC4801190; doi:10.1371/journal.pone.0151736)
Supplement: S1 File — (DOCX) [file pone.0151736.s003.docx]

**Supplementary Materials and Methods**

All reagents were purchased from Sigma Aldrich (St Louis, MO) unless otherwise stated.

**Western blotting**

Proteins from ECM scaffold were extracted in 4M GnHCl, 50 mM sodium acetate pH 5.8, 1X protease inhibitor. All supernatants were then dissolved in RIPA buffer containing protease inhibitors. Protein concentrations were determined with the Bio-Rad assay reagent (Bio-Rad Laboratories, Hercules, CA). Proteins were separated on Tris-Acetate 3−8% and Bis-Tris 4−12% gradient polyacrylamide gels (Invitrogen) and transferred to a nitrocellulose membrane. Membranes were then blocked with 0.5% Tween-PBS containing 5% nonfat dried milk and incubated overnight with the primary antibodies (albumin, calnexin, and collagen alpha-1(I) chain, Santa Cruz Biotechnology (Santa Cruz, USA)) followed by incubation with HRP-conjugated species-specific secondary antibodies and enhanced chemiluminescence reaction using ECL Plus Western Blotting Detection System (GE Healthcare, Chalfont St Giles, U.K.).

**Scanning Electron Microscopy**

Decellularized liver samples were fixed in 2% paraformaldehyde and 2.5% glutaraldehyde in 0.2 M sodium phosphate buffer for 24 h at room temperature. The fixed samples were washed three times with fresh buffer and dehydrated with a series of ethanol solutions of increasing concentration, 70%, 80%, 90% and 100%. The samples were covered with a 30 nm layer of Chromium using a Quorum Q150T ES sputter. The Electron Microscopy analysis has been performed using a FESEM Zeiss Auriga 405, operating with an acceleration voltage of 5 keV and working distance of 5 millimeters.

**Nano LC-MS/MS**

For proteomics analysis ECM scaffolds purified from human biopsy (length 1 cm) were resuspend in 40 uL of NH_4_HCO_3_ 50mM, urea 2M solution and digested 5uL of tripsin solution [0,2 ug/uL] overnight at 37°C. Peptides were reduced in 10mM Dithiothreitol (DTT) at 56°C for 30 minutes. Alkylations were performed in 55mM Iodoacetamide (IAA) at room temperature for 20 minutes in the dark. Peptides were then desalted and filtered through a C18 microcolumn ZipTip, and eluted from the C18 bed using 10 μL of 80% acetonitrile/0.1% formic acid. The organic component was removed by evaporation in a vacuum centrifuge and peptides were resuspended in 50 µl of 0.1% formic acid and analysed by nano LC-MS/MS by an Ultimate 3000 system (Dionex, Sunnyvale, CA, USA) equipped with a splitting cartridge for nanoflow and connected on-line *via* a nano-ESI source (Thermo-Fisher Scientific, Waltham, Massachussetts, USA) to an LTQ-Orbitrap XL mass spectrometer (Thermo-Fisher Scientific).

Twenty microliters of each peptide mixture were automatically loaded onto a pre-column cartridge for peptide concentration (Acclaim^®^ PepMap^™^ μ-Precolumn, 300μm x 1 mm, Dionex). The pre-column was washed with 4% of acetonitrile containing 0.1% formic acid at a flow rate of 20 µl/min for 4 min. Peptides were separated on a 15 cm long analytical column (PicoFrit^®^360 μm o.d., 75 μm i.d., 15μm tip i.d., New Objective, Woburn, MA, USA) packed with reverse phase resin (Magic C18AQ 200 Å pore size C18, 5 μm i.d.). The mobile phase A was 0.1% formic acid and mobile phase B was 0.1% formic acid in acetonitrile. The multistep elution gradient was the following: from 5% to 40% of phase B within 120 min, from 40% to 60% of B in 10 min, from 60% to 80% of B in 5 min at a constant flow rate of 300 nl/min.

Eluted peptides were electrosprayed directly into the mass spectrometer with an ESI voltage of 1.9 kV. MS data were acquired in a positive mode in the Orbitrap in FTMS mode over 300-2000 *m/z* range with resolution 30,000 at *m/z*=400, with an automatic gain control (AGC) target of 1×10^6^ ions, and the maximal injection time of 1000 ms. Tandem mass spectra were acquired into the linear ion trap quadrupole (ITMS) by data-dependent mode with the Excalibur software (Thermo-Fisher Scientific), selecting the five most intense ions with charge states ≥ 2 detected *per* survey scan by FTMS, through collision-induced dissociation (CID), and analysing the resulting fragments in the linear trap (LTQ). For MS/MS scanning, the minimum MS signal was set to 500, activation time to 30 ms, target value to 10,000 ions, and injection time of 100 ms. All MS/MS spectra were collected using a normalized collision energy of 35% and an isolation window of 2 Th. To avoid redundant sequencing of the most abundant peptides, dynamic exclusion was enabled with a repeat count of 1, a repeat duration of 30 s, an exclusion list size of 300 and an exclusion duration of 90 s.

Proteins were automatically identified using the proteomics software package MaxQuant (version 1.3.5). Tandem mass spectra were searched against the *Homo sapiens* dataset of UniprotKB database (Release: March 2015; 68,511 sequences). Trypsin was selected as cleavage enzyme. A maximum of 2 missed cleavages was allowed. Mass tolerance for FTMS and for ITMS measurements were respectively set to 20 ppm and 0.5 Da. The False Discovery Rate (FDR) for proteins and peptides identification was set to 1%. Carbamidomethylation of cysteine was set as a fixed modification. The following variable modifications were used for both identification and quantification: oxidation of methionine; lysine acetylation; proline and lysine hydroxylation; serine, tyrosine and threonine phosphorylation. Minimum peptides length was set to 7 amino acids. For protein quantification was enabled the label free quantification (LFQ) algorithm. Searches were also implemented querying dataset of commonly detected contaminants in proteomics as well as the reverse decoy database generated by the Andromeda search engine.

**Label free quantitative analysis**

The comparison among the four fibrotic stages were performed using the LFQ protein intensities calculated by MaxQuant (http://www.maxquant.org). Quantitative ratio for each protein was estimated using F1 fibrotic stage as the reference sample. Each fibrotic stage was analysed in triplicate. One-way ANOVA for multiple sample was performed using the freely available Perseus software (http://www.maxquant.org) after log2 transformation of the intensity data; a p-value ≤ 0.05 was accepted as significant.
